# Cost analysis of laparoscopic total versus open total gastrectomy in gastric cancer

**DOI:** 10.1007/s00423-024-03562-y

**Published:** 2025-01-08

**Authors:** Max M. Maurer, Sebastian Knitter, Axel Winter, Ramin Raul Ossami Saidy, Eva M. Dobrindt, Philippa Seika, Paul V. Ritschl, Jonas Raakow, Judith Reinus, Johann Pratschke, Christian Denecke

**Affiliations:** 1 Department of Surgery, Charité – Universitätsmedizin Berlin, Corporate Member of Freie Universität Berlin and Humboldt-Universität zu Berlin, Campus Charité Mitte and Campus Virchow-Klinikum, Augustenburger Platz 1, 13353 Berlin, Germany; 2 Berlin Institute of Health at Charité – Universitätsmedizin Berlin, BIH Biomedical Innovation Academy, BIH Charité Clinician Scientist Program, Charitéplatz 1, 10117 Berlin, Germany; 3 Department of Medical Controlling, Charité - Universitätsmedizin Berlin, Corporate Member of Freie Universität Berlin and Humboldt-Universität zu Berlin, Charitéplatz 1, 10117 Berlin, Germany

**Keywords:** Upper gastrointestinal surgery, Laparoscopic surgery, Total gastrectomy, Cost analysis

## Abstract

**Purpose:**

Despite ongoing discussions concerning clinical equivalence of laparoscopic total gastrectomy (LTG) compared to open total gastrectomy (OTG) in gastric cancer (GC) surgery, complementary evidence regarding financial implications is sparse. The aim of this study was to compare hospital associated expenses and perioperative outcomes between both approaches.

**Methods:**

Clinicopathological and financial data from 80 consecutive GC patients undergoing LTG or OTG between 2015 and 2022 were investigated. Patient baseline characteristics, perioperative results, long-term outcomes and financial expenses up to 30 days after discharge were compared. A binary logistic regression model to identify individual cost drivers was performed.

**Results:**

LTG was associated with significantly prolonged operating time (281 min vs. 245 min, *p* < 0.02). However, LTG demonstrated a trend towards lower total (18,708 € vs. 22,810 €, *p* = 0.11) and median daily (1,516 € vs. 1,721 €, *p* = 0.25) expenses, yet not reaching statistical significance. Decreased ICU costs emerged as the greatest single cost reducer in LTG (962 € vs. 2,147 €, *p* = 0.10). Hospital length of stay ≥ 15 days was the only independent cost driver for increased expenses (HR [95% CI] = 13,2 [3.0-58.9], *p* < 0.01). Ultimately, patients undergoing LTG and OTG demonstrated similar outcomes in terms of perioperative morbidity (*n* = 8, 13% vs. *n* = 3, 17%, *p* = 0.70), median number of resected lymph nodes (*n* = 32 vs. *n* = 33, *p* = 0.72), absence of 90-day mortality, and long-term survival (*p* = 0.47).

**Conclusion:**

Although typically involving longer operating times, LTG tends to be linked with decreased hospital costs, yet not reaching statistical significance. The ongoing establishment of LTG seems not to pose additional financial burdens for surgical departments.

**Supplementary Information:**

The online version contains supplementary material available at 10.1007/s00423-024-03562-y.

## Introduction

Gastrectomy remains the only potentially curative therapeutic option for patients with advanced gastric cancer, still accounting for more than 700,000 deaths globally per year [1–3]. Open total gastrectomy (OTG) has been the standard surgical approach for almost a century [4]. However, following its first introduction for early-stage distal cancer in 1994, minimal invasive techniques have been extended from initially only partial resections to laparoscopic total gastrectomies (LTG) [5].

In contrast to partial resections, LTG is not yet fully implemented as the standard surgical approach in national and international oncologic guidelines but rather recommended as an option in high-volume centers with sufficient expertise [6, 7]. However, LTG has proven beneficial for patients in terms of minimized surgical trauma, decreased risk of wound infections [8], shorter hospital stays [9, 10], and accelerated postoperative recovery [8, 9]. Moreover, growing evidence indicates comparable results between OTG and LTG regarding oncological long-term outcomes [9, 11, 12], disease-free survival [13, 14], and quality of life [15]. Subsequently, LTG has been increasingly adopted as the standard approach in high-volume centers within the last decade.

Considering this transition, complementary financial considerations for health care providers gain increasing significance with surgical departments being affected in the first place. Yet, in contrast to other surgical domains, substantial evidence regarding financial disparities between OTG and LTG for performing departments is lacking. Corresponding research comparing financial implications between laparoscopic and open procedures in the fields of colorectal [16], hepatic [17, 18], and pancreatic surgery [19] suggest higher intraoperative expenses associated with minimal invasive techniques, that yet get outbalanced by reduced costs in terms of complication management and overall length of hospital stays. After all, not all patients may be viable candidates for LTG due to complicating preconditions. Therefore, a better understanding of cost disparities between OTG and LTG might allow for more accurate budgeting in health care systems based on diagnose-related groups (DRGs) in the future.

Given the sparse literature, this study aims to compare perioperative cost expenses between oncologic OTG and LTG in a German tertiary hospital. Additionally, patient and treatment related cost drivers shall be investigated. Secondary outcomes being evaluated include the comparison of perioperative morbidity and mortality associated with each approach.

## Materials and methods

### Study design

This was a retrospective single-center study enrolling patients who underwent OTG or LTG for gastric cancer (GC) or carcinoma of the gastroesophageal junction (GEJ) between 2015 and 2022 at the Department of Surgery, Campus Charité Mitte | Campus Virchow-Klinikum, Charité – Universitätsmedizin Berlin. Demographic patient characteristics, comorbidities, pre-operative laboratory values, disease-related factors, perioperative parameters, outcome measurements and hospital incurred financial expenses up to 30 days after discharge were considered for analysis. Complications were graded according to the Clavien-Dindo-classification and any complication ≥ 3a was considered as major morbidity. Hand-assisted LTG with D2 lymphadenectomy is the standard surgical approach at our center. Decision for OTG is based on individual case-by-case evaluation. Approval by the ethics committee of the Charité – Universitätsmedizin Berlin (EA2/213/23) was granted.

### Analysis of financial expenses

A standardized cost matrix was applied to categorize financial data into 12 distinct subdomains: (1) surgery, (2) anesthesia, (3) intensive care unit (ICU), (4) dialysis, (5) care on the normal ward, (6) laboratory tests, (7) cardiology, (8) radiology, (9) endoscopy, (10) other diagnostics, (11) other therapeutics including physiotherapy, and (12) patient admission. Costs for medical and non-medical staff, consumables, and logistics were subsumed in each section. Additionally, total and daily adjusted costs per patient per stay were calculated. All numbers are presented in Euro (€).

### Statistical analysis

Patient, tumor, perioperative, and financial data were compared between OTG and LTG. Continuous variables were depicted as medians with interquartile range (IQR) and analyzed using the Mann-Whitney U test. Categorical variables were presented as frequencies and juxtaposed using the Chi-square or Fisher’s exact test depending on suitability. Elevated costs were delineated as expenses per case surpassing the 75th percentile of the entire cohort.

Factors correlated with escalated costs were identified employing a binary logistic regression model. Outcomes were articulated as hazard ratios (HR) and 95% confidence intervals (CI) after multivariate analysis of all parameters exhibiting *p* < 0.1 in univariate analysis (Chi-square or Fisher’s exact test). Statistical significance was defined at *p* < 0.05.

All statistical analyses were performed using the SPSS software package for Mac OS, version 27 (IBM, Armonk, NY, USA).

## Results

### Patient baseline characteristics

A total of 227 gastrectomy cases performed between January 2015 and December 2022 were identified. After excluding cases conducted for benign conditions, non-gastric cancer malignancies, subtotal gastrectomies, procedures involving intraoperative chemotherapy, robotic-assisted surgeries, conversions to open surgery, and extended transhiatal gastrectomies, 88 patients with locally advanced GC and GEJ cancer receiving total gastrectomy remained eligible for analysis. The majority (*n* = 66, 75%) underwent LTG and 22 (25%) patients received OTG. Three patients underwent OTG in an emergency setting and were hence not considered for analysis. Moreover, five patients received extended, non-tumor related diagnostics prior to surgery during their inpatient stay and were thus excluded. Restaging endoscopy and abdominothoracic computertomography was performed in around one quarter of patients in both cohorts during their hospitalization and resultant costs equally included (LTG: *n* = 18, 28%; OTG: *n* = 4, 22%). Individual decisions to opt for OTG included a history of major abdominal surgery, tumor seize, gastric stent implantation, or extensive cardiopulmonary risk factors.

A detailed description of patient characteristics is given in Table 1. The majority of patients were male (*n* = 51, 63%) and median age was 66 (53–73) years with no statistical significant difference between the procedural cohorts (*p* = 0.17). There was no significant difference regarding Body Mass Index (BMI; LTG: 24.5 [22.2–28.1] vs. OTG: 24.6 [22.3–28.3]; *p* = 0.81) nor Eastern Cooperative Oncology Group (ECOG) with most patients presenting at ECOG 0 (*p* = 0.83). Patients undergoing OTG showcased significantly higher American Society of Anesthesiology Score (ASA) groups (*p* = 0.04). Likewise, they presented with a higher Charlson Comorbidity Index (CCI; OTG: 9, [6.5–11.0] vs. LTG: 6.5 [3.0–11.0]), however, this difference did not reach statistical significance (*p* = 0.07). The most common comorbidity in both groups was arterial hypertension (LTG: *n* = 34, 55%; OTG: *n* = 10, 56%; *p* = 0.96). Most patients received neoadjuvant therapy (LTG: *n* = 56, 90%; OTG: *n* = 14, 94%; *p* = 1.00) with an equal number of chemotherapy cycles per patient. There was no significant difference in the tumor entity distribution with diffuse carcinoma being more prevalent in both groups (LTG: *n* = 34, 55%; OTG: *n* = 11, 61%; *p* = 0.64).

**Table 1:** Clinicopathological data of 80 patients who underwent LTG or OTG for GC and cancer of the GEJ

Characteristic	Overall, *N* = 80^*1*^	LTG, *N* = 62^*1*^	OTG, *N* = 18^*1*^	*p*-value^2^
Gender				0.072
female	30 (38%)	20 (32%)	10 (56%)	
male	50 (63%)	42 (68%)	8 (44%)	
Age	66 (53, 73)	65 (53, 71)	70 (54, 77)	0.172
BMI	24.5 (22.2, 28.1)	24.5 (22.2, 28.1)	24.6 (22.3, 28.3)	0.815
ECOG				0.833
0	65 (81%)	49 (79%)	16 (89%)	
1	13 (16%)	11 (18%)	2 (11%)	
2	2 (2.5%)	2 (3.2%)	0 (0%)	
ASA				**0.035**
I	3 (3.8%)	3 (4.8%)	0 (0%)	
II	28 (35%)	26 (42%)	2 (11%)	
III	47 (59%)	32 (52%)	15 (83%)	
IV	2 (2.5%)	1 (1.6%)	1 (5.6%)	
CCI	8.0 (4.0, 11.0)	6.5 (3.0, 11.0)	9.0 (6.5, 11.0)	0.069
Diabetes	14 (18%)	11 (18%)	3 (17%)	1.00
Coronary heart disease	8 (10%)	8 (13%)	0 (0%)	0.188
Arterial hypertension	44 (55%)	34 (55%)	10 (56%)	0.957
Pulmonary disease	11 (14%)	9 (15%)	2 (11%)	1.00
Neoadjuvant treatment	72 (91%)	56 (90%)	16 (94%)	1.00
Number of CC	4.00 (4.00, 4.00)	4.00 (4.00, 4.00)	4.00 (4.00, 4.00)	0.466
pT stage				0.093
0	6 (7.5%)	4 (6.5%)	2 (11%)	
1a	8 (10%)	8 (13%)	0 (0%)	
1b	14 (18%)	13 (21%)	1 (5.6%)	
2	13 (16%)	10 (16%)	3 (17%)	
3	29 (36%)	22 (35%)	7 (39%)	
4a	9 (11%)	5 (8.1%)	4 (22%)	
4b	1 (1.3%)	0 (0%)	1 (5.6%)	
pN stage				0.051
0	48 (60%)	39 (63%)	9 (50%)	
1	17 (21%)	13 (21%)	4 (22%)	
2	6 (7.5%)	5 (8.1%)	1 (5.6%)	
3a	6 (7.5%)	5 (8.1%)	1 (5.6%)	
3b	3 (3.8%)	0 (0%)	3 (17%)	
Lymphangiosis carcinomatosa	24 (30%)	14 (23%)	10 (56%)	**0.007**
Venous invasion	4 (5.0%)	2 (3.2%)	2 (11%)	0.217
Pathology				0.637
diffuse	45 (56%)	34 (55%)	11 (61%)	
intestinal	35 (44%)	28 (45%)	7 (39%)	
Grading				**0.083**
X	6 (7.5%)	4 (6.5%)	2 (11.1%)	
2	14 (17.5%)	14 (22.6%)	0 (0%)	
3	60 (75.0%)	44 (71.0%)	16 (88.9%)	

^*1*^n (%); Median (IQR)

^*2*^Pearson’s Chi-squared test; Wilcoxon rank sum test; Fisher’s exact test

BMI, Body Mass Index; ECOG, Eastern Cooperative Oncology Group; ASA, American Society of Anesthesiologists; CCI, Charlson Comorbidity Index; CC, chemotherapy cycles

### Perioperative outcomes

Perioperative outcomes are shown in Table 2. OTG showed significantly shorter operating time (245 min [214, 266]) compared to LTG (281 min [245, 328]; *p* = 0.02). Adversely, LTG patients tended to spend fewer days on the ICU (1 day [1, 2]) compared to the OTG group (2 days [1, 4]), however, this difference did not reach statistical significance (*p* = 0.13). Moreover, LTG patients exhibited a shorter length of hospital stay (12 days [9, 15]), compared to OTG (13 days [10, 15]), although again not reaching statistical significance (*p* = 0.56). Within both groups, approximately one out of ten patients required ICU re-admittance following primary transferal to the normal ward after standardized post-operative ICU surveillance (LTG: *n* = 6, 9.7%; OTG: *n* = 2, 11%; *p* = 1.00). Of particular note, LTG patients showed a higher rate of postoperative pneumonia (*n* = 9, 15%) compared to only one episode in the OTG group (5.6%), yet not reaching statistical significance (*p* = 0.44). There was no difference regarding the occurrence of anastomotic leakage (LTG: *n* = 4, 6.5% vs. OTG: *n* = 1, 5.6%; *p* = 1.00) nor stenosis or pylorospasm requiring bouging or botox-injection, respectively. Out of the five cases with confirmed anastomotic leakage, the majority was treated primarily endoscopically as 40% (*n* = 2) were managed with endosponge therapy and a further 40% (*n* = 2) received a combined stent-sponge treatment. Only one case required primary surgical revision. Likewise, four patients with postoperative anastomotic constrictions underwent endoscopic bougienage. Although 10% (*n* = 8) of patients required re-surgery, the majority of these procedures were minor interventions, such as port removals due to device infection and surgical tracheostomies. Hence, only three cases involved more significant revisions, which included one case of abdominal flushing, one case with oversewing of the anastomosis, and one cardiothoracic intervention. Finally, overall complication rates (LTG: *n* = 13, 21% vs. OTG: *n* = 3, 17%) as well as major perioperative morbidity (LTG: *n* = 8, 13% vs. OTG: *n* = 3, 17%) did not differ significantly between the two cohorts (*p* = 1.00 and *p* = 0.70, respectively). Two patients after LTG (3.2%) and one patient receiving OTG (5.6%) were re-admitted within 30 days after primary discharge due to surgical related complications. There was no mortality in neither of the groups in terms of in-house, 30-day or 90-day mortality.

**Fig. 1:**
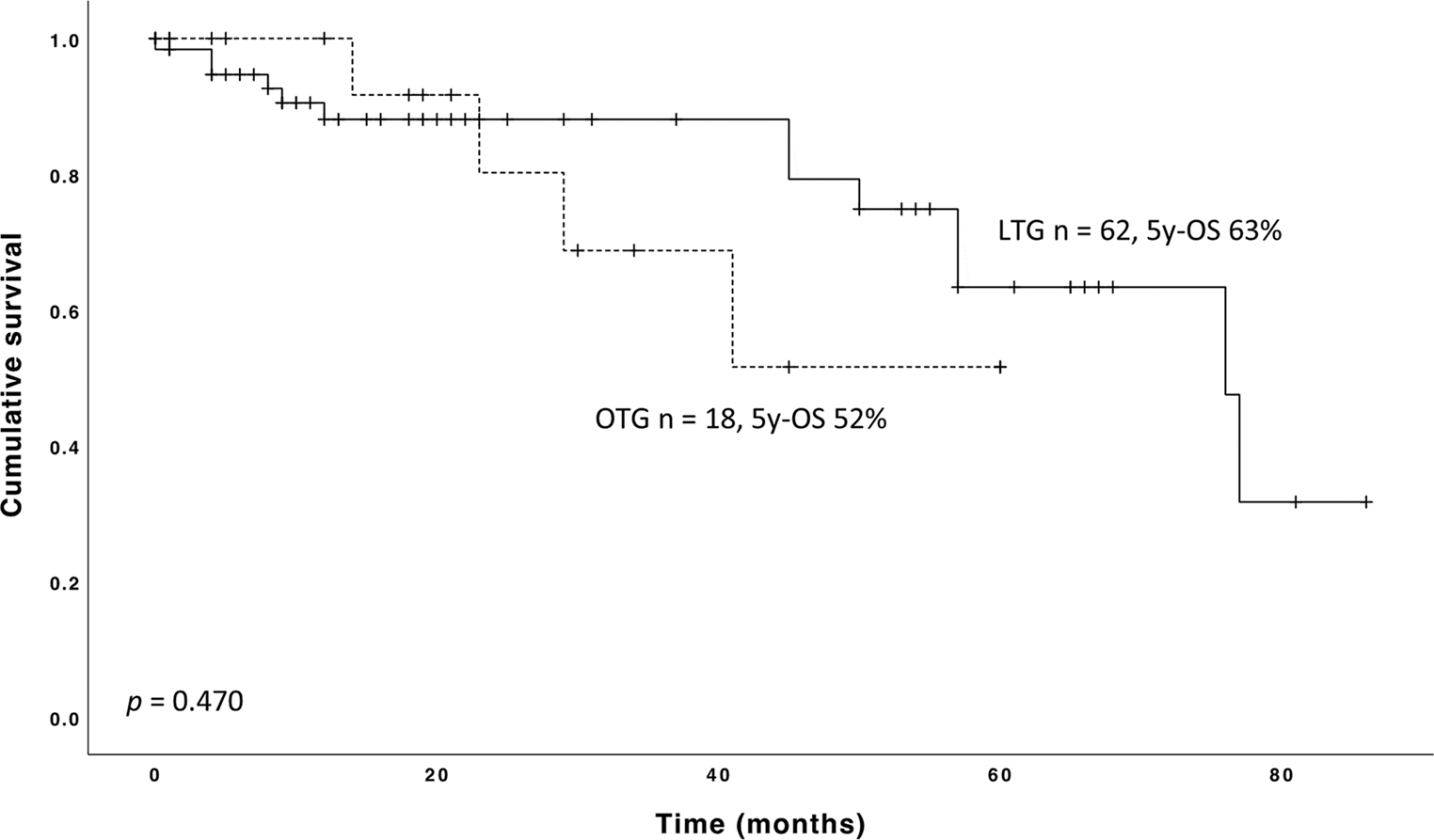
Kaplan-Meier-plot of 80 patients who underwent oncologic LTG or OTG for GC or cancer of the GEJ

Finally, both groups showed equal yields of resected lymph nodes (LTG: 32 [26, 41]; OTG: 33 [28, 40]; *p* = 0.72). While none of the LTG patients showcased positive resection margins (*n* = 62, 0%), this was the case in 3 OTG patients (17%, *p* = 0.10). However, tumor infiltration of the duodenum with no opportunity for further resection was noted intraoperatively. Although LTG patients tend to show a reduced survival compared to OTG within the 12 months following primary resection, Kaplan-Meier analysis revealed comparable long-term survival rates (*p* = 0.47; Fig. 1).

**Table 2:** Perioperative data of 80 patients who underwent oncologic LTG or OTG for GC or cancer of the GEJ

Characteristic	Overall, *N* = 80^*1*^	LTG, *N* = 62^*1*^	OTG, *N* = 18^*1*^	*p*-value^2^
Length of surgery, min	273 (240, 321)	281 (245, 328)	245 (214, 266)	**0.020**
Number of administered RBCC				0.731
0	75 (94%)	58 (94%)	17 (94%)	
1	3 (3.8%)	2 (3.2%)	1 (5.6%)	
2	2 (2.5%)	2 (3.2%)	0 (0%)	
Number of resected lymph nodes	32 (26, 41)	32 (26, 41)	33 (28, 40)	0.721
Number of positive lymph nodes	0.0 (0.0, 2.0)	0.0 (0.0, 1.0)	0.5 (0.0, 2.8)	0.202
Positive resection margins	3 (3.8%)	0 (0%)	3 (17%)	**0.010**
Need for re-operation	8 (10%)	5 (8.1%)	3 (17%)	0.370
Pneumonia	10 (13%)	9 (15%)	1 (5.6%)	0.442
Anastomotic leakage	5 (6.3%)	4 (6.5%)	1 (5.6%)	1.00
Anastomotic stenosis	3 (3.8%)	3 (4.8%)	0 (0%)	1.00
Length of stay on ICU, days	1 (1, 3)	1 (1, 2)	2 (1, 4)	0.131
Readmission to ICU	8 (10%)	6 (9.7%)	2 (11%)	1.00
Length of hospital stay, days	12 (9, 15)	12 (9, 15)	13 (10, 15)	0.563
Overall morbidity	16 (20%)	13 (21%)	3 (17%)	1.00
Major morbidity	11 (14%)	8 (13%)	3 (17%)	0.704
30-day mortality	0 (0%)	0 (0%)	0 (0%)	-
90-day mortality	0 (0%)	0 (0%)	0 (0%)	-

^*1*^Median (IQR); n (%)

^*2*^Wilcoxon rank sum test; Fisher’s exact test

RBCC = red blood cell concentrations

### Cost analysis and factors associated with increased costs

A complete description regarding all cost domains is given in Table 3. LTG was associated with both lesser total (18,708 € [15,904, 22,998]) and day adjusted (1,516 € [1,338, 1,828]) costs compared to OTG (22,810 € [18,519, 25,882] and 1,721 € [1,422, 2088], respectively). However, this reduction did not reach statistical significance (*p* = 0.124 and *p* = 0.268, respectively). OTG only revealed smaller costs in the domains of surgery (OTG: 6,859 € [5,517, 7,659] vs. LTG 7,036 € [6,129, 8,528]; *p* = 0.49) and radiology (OTG: 161 € [50, 364] vs. LTG 368 € [168, 580]; *p* = 0.08), while LTG economically outperformed the open approach in all remaining fields. While only higher laboratory costs between the two cohorts achieved statistical significance (OTG: 2,168 € [1,487, 2,523] vs. LTG 1,467 € [1,028, 2,523]; *p* = 0.03), expenses for ICU showed the most pronounced difference between both cohorts (OTG: 2,147 € [996, 5,718] vs. LTG: 962 € [736, 2,727]; *p* = 0.10).

### Cost analysis and factors associated with increased costs

A complete description regarding all cost domains is given in Table 3. LTG was associated with both lesser total (18,708 € [15,904, 22,998]) and day adjusted (1,516 € [1,338, 1,828]) costs compared to OTG (22,810 € [18,519, 25,882] and 1,721 € [1,422, 2088], respectively). However, this reduction did not reach statistical significance (*p* = 0.124 and *p* = 0.268, respectively). OTG only revealed smaller costs in the domains of surgery (OTG: 6,859 € [5,517, 7,659] vs. LTG 7,036 € [6,129, 8,528]; *p* = 0.49) and radiology (OTG: 161 € [50, 364] vs. LTG 368 € [168, 580]; *p* = 0.08). Of note, those surgical costs do also include expenses of additional re-surgeries, which has to be considered for three patients with major procedures as outlined above. In contrast, LTG economically outperformed the open approach in all remaining fields. While only higher laboratory costs between the two cohorts achieved statistical significance (OTG: 2,168 € [1,487, 2,523] vs. LTG 1,467 € [1,028, 2,523]; *p* = 0.03), expenses for ICU showed the most pronounced difference between both cohorts (OTG: 2,147 € [996, 5,718] vs. LTG: 962 € [736, 2,727]; *p* = 0.10). To further investigate the parameters associated with increased laboratory costs, an additional multivariate analysis was performed (Table 1 in Supplementary Material). Specifically, we examined which patient characteristics were linked to laboratory costs exceeding the 75th percentile. Univariate analysis initially identified several significant factors, including diabetes (*p* = 0.04), neoadjuvant therapy (*p* = 0.01), lymphangiosis carcinomatosis (*p* = 0.01), anastomotic leakage (*p* = 0.01), ICU stay longer than 3 days (*p* = 0.04), hospital stay longer than 15 days (*p* = 0.01), ICU readmission (*p* < 0.01), and major morbidity (*p* = 0.02). However, the subsequent regression analysis highlighted ICU readmission as an independent parameter.

**Table 3:** Financial data of 80 patients who underwent oncologic LTG or OTG for GC or cancer of the GEJ

Characteristic	Overall, *N* = 80^*1*^	LTG, *N* = 62^*1*^	OTG, *N* = 18^*1*^	*p*-value^2^
Normal ward	4,835 (3,614, 6,823)	4,763 (3,508, 6,499)	5,362 (4,226, 7,482)	0.220
ICU	1,028 (792, 3,803)	962 (736, 2,727)	2,147 (996, 5,718)	0.103
Dialysis	13,854 (7,593, 20,115)	13,854 (7,593, 20,115)	NA (NA, NA)	
Surgery	6,995 (6,024, 8,519)	7,036 (6,129, 8,528)	6,859 (5,517, 7,659)	0.486
Anesthesia	2,320 (1,900, 2,853)	2,303 (1,867, 2,802)	2,451 (1,915, 3,035)	0.504
Endoscopy	318 (163, 503)	318 (171, 539)	343 (133, 381)	0.542
Radiology	344 (128, 530)	368 (168, 580)	161 (50, 364)	0.076
Laboratory	1,567 (1,181, 2,215)	1,467 (1,028, 2,077)	2,168 (1,487, 2,523)	**0.033**
Other diagnostics	114 (41, 158)	96 (29, 145)	122 (101, 283)	0.076
Other therapeutics	222 (126, 364)	213 (126, 311)	318 (143, 437)	0.189
Admission	59 (48, 67)	48 (48, 67)	59 (59, 67)	0.208
Total costs	19,085 (16,032, 24,378)	18,708 (15,904, 22,998)	22,810 (18,519, 25,882)	0.105
Daily costs	1,537 (1,363, 1,861)	1,516 (1,338, 1,828)	1,721 (1,422, 2,088)	0.247

^*1*^Median (IQR)

^*2*^Wilcoxon rank sum test

In order to discern specific factors driving increased expenses, an additional analysis encompassing patient, tumor, surgery and postoperative parameters was conducted. Elevated costs were defined as cumulative expenditures surpassing the 75th percentile of the entire cohort, totaling 24,625 € (Table 4). While neither patient nor tumor related characteristics proved to be significant cost drivers, only several postoperative complications such as pneumonia (*p* < 0.01), need for re-operation (*p* < 0.01), anastomotic leakage (*p* < 0.01), readmission to ICU (*p* < 0.01), major morbidity (*p* < 0.01), and ICU (*p* < 0.01), as well as hospital length of stays exceeding the 75th percentile (*p* = 0.01), emerged as significant parameters individually. However, multivariate analysis revealed that only a hospital stay exceeding ≥ 15 days was the decisive factor contributing to increased costs.

**Table 4:** Multivariate analysis of factors associated with increased total costs in the most cost-intensive 20 patients (beyond 75th percentile of the entire cohort) who underwent OTG or LTG for EC or cancer of the GEJ

Parameters	UV			MV	
	< 24,625 €/case (*n* = 60)^1^	≥ 24,625 €/case (*n* = 20)^1^	*P* ^2^	HR (95% CI)	*P* ^3^
Male sex	37 (62%)	13 (65%)	0.790		
Age ≥ 65 years	29 (48%)	13 (65%)	0.301		
BMI ≥ 30 kg/m^2^	10 (17%)	2 (10%)	0.454		
ASA score ≥ 3	22 (37%)	9 (45%)	0.508		
CCI ≥ 11^4^	17 (28%)	8 (40%)	0.330		
Diabetes	10 (17%)	4 (20%)	0.741		
Coronary heart disease	4 (7%)	4 (20%)	0.102		
Arterial hypertension	33 (55%)	11 (55%)	> 0.999		
Pulmonary disease	7 (12%)	4 (20%)	0.454		
Neoadjuvant treatment	56 (95%)	16 (80%)	**0.064**		NS
T stage ≥ 3	31 (52%)	10 (50%)	0.897		
Nodal positive disease	37 (62%)	11 (55%)	0.598		
Lymphangiosis carcinomatosa	17 (28%)	7 (35%)	0.573		
Venous invasion	4 (7%)	0 (0%)	0.567		
Positive resection margins	2 (3%)	1 (5%)	> 0.999		
Length of surgery ≥ 321 min^4^	13 (22%)	8 (40%)	0.107		
Need for re-operation	2 (3%)	6 (30%)	**0.003**		NS
Pneumonia	3 (5%)	7 (35%)	**0.002**		NS
Anastomotic insufficiency	0 (0%)	5 (25%)	**< 0.001**		NS
Anastomotic stenosis	1 (2%)	2 (10%)	0.153		
Length of ICU stay ≥ 3 days^4^	12 (20%)	10 (50%)	**0.009**		NS
Readmission to ICU	1 (2%)	7 (35%)	**< 0.001**		NS
Length of hospital stay ≥ 15 days^4^	6 (10%)	15 (75%)	**< 0.001**	13.2 (3.0-58.9)	**0.001**
Major morbidity	2 (3%)	9 (45%)	**< 0.001**		NS

^1^ n (%)

^2^ Fisher’s exact test; Pearson’s Chi-squared test

^3^ Binary logistic regression

^4^ 75th percentile of all patients

UV, univariate analysis; MV, multivariate analysis; BMI, body mass index; ASA, American Society of Anesthesiologists; CCI, Charlson comorbidity Index; ICU, intensive care unit; NS, not significant; n/a, not applicable

## Discussion

Surgery constitutes for up to one third of all healthcare expenses in Western countries [20], emphasizing the pivotal need for critical evaluation and precise identification of cost drivers within this domain. Consequently, evidence regarding financial implications associated with new surgical techniques is of crucial importance to improve and ensure comprehensive planning reliability for departments aiming to implement novel techniques.

Minimal-invasive total gastrectomy has seen a considerable surge during the last decade [21]. This transition has been accompanied by numerous comparative studies predominantly investigating perioperative outcomes and oncologic quality as the primary criteria in determining the optimal surgical approach. In our study, OTG and LTG demonstrated comparable short- and long-term clinical results. There were no differences regarding inhouse-, 30-day or 90-day mortality as no patient in neither of the cohorts deceased within these observational periods. Likewise, no significant difference regarding overall complication rates or perioperative major morbidity was observed. Notably, we observed a higher occurrence of pneumonia after LTG. This contrasts with previous studies, which have shown that laparoscopic approaches generally reduce the risk of pneumonia for various procedures, including total and distal gastrectomies [10, 22]. Possible explanations for this observation include reduced pain, less impaired diaphragmatic function, and a reduced pro-inflammatory cytokine response in minimally invasive compared to open surgery [22]. However, the opposite finding in our cohort did not reach statistical significance. Both procedures furthermore achieved comparable lymph node harvest in terms of procedural oncologic quality. LTG patients showcased a slightly higher long-term survival, however, this difference did not reach statistical significance.

Overall, our findings support previous results suggesting comparable outcomes between OTG and LTG in terms of safety, oncologic surgical quality and long-term outcomes. In contrary to a profound number of clinical comparisons, evidence regarding financial implications between OTG and LTG is sparse. In our study, LTG revealed overall lower costs for both total and day adjusted expenses in comparison to OTG. In accordance with previous studies, LTG was associated with significant longer operating time [9, 23], leading to increased expenses in this domain in the first place. However, patients tend to spend less time on the ICU and demonstrated an overall shorter total length of in-hospital stay. Moreover, OTG was associated with significantly higher laboratory expenses during postoperative care. Interestingly, costs for anesthesia were not elevated in LTG compared to OTG patients despite longer operation time. This observation may be attributed to the requirement for more intricate anesthesiological support in OTG patients as indicated by significantly higher ASA groups within this cohort. No specific patient or tumor related parameter emerged as individual cost driver. While various post-operative complications were identified as univariate contributors to upraised costs, only prolonged hospital stay persisted as a significant factor following multivariate analysis. However, it’s worth noting that this outcome could potentially be influenced by the limited sample size.

Only four studies have previously taken the economic impact between OTG and LTG into account. Unlike our findings, financial analysis alongside the prospective, multicenter LOGICA trail found that higher intraoperative costs in LTG were only partially compensated by reduced postoperative expenses [15]. A primary reason for the discrepancy arises from a more pronounced difference in operating time between OTG and LTG, which was 20.4% among the LOGICA trail compared to only 13.7% in our analysis. This variance could be attributed to the selective performance of OTG in patients with complicating preconditions in our study, whereas the LOGICA trial adhered to randomized assignment. Yet, our patient selection based on clinical criteria may rather reflect the clinical reality in that laparoscopic surgery is offered to all patients unless they are too sick and need open surgery. After all, reduced ICU expenses were consistently identified as the most important cost-saving factor for LTG in both analyses.

An earlier study utilizing US health care data found no difference in hospital treatment groups between minimal-invasive and open gastrectomy, however lacking a detailed subanalysis to differentiate among partial and total gastrectomy [24]. Moreover, a single-center study from the Netherlands demonstrated higher costs of disposables and theater time associated with LTG, which could be offset by overall shorter length of stay, though the subsets of OTG and LTG were rather limited (9 vs. 14 patients) [25]. Finally, a recent retrospective study conducted at a single center in China revealed significantly escalated surgical expenses but ultimately diminished hospitalization costs for patients undergoing LTG, aligning with our findings [26]. Besides longer time of surgery, the authors also highlight the role of increased material costs due to the need for imports of laparoscopic disposals, which underscores challenges in cost comparability on an international scope.

In summary, our findings indicate that LTG achieves comparable outcomes in terms of safety and oncological efficacy, alongside favorable economic implications when contrasted with OTG. Notably, a shorter overall hospitalization duration and particularly reduced ICU stays emerge as crucial factors in cost reduction within this realm. Conversely, patients individually necessitating OTG due to complicating preconditions do not impose a substantial financial strain on surgical departments, as the increased costs did not achieve statistical significance. However, arriving at a conclusive assessment remains challenging. Despite constraints in generalizability arising from budgetary variations across diverse health care systems, cost-effectiveness should also consider patient-related factors such as quality-adjusted life years (QUALYs). Lastly, the comprehensive financial implications must also encompass direct longterm expenditures for ensuing healthcare providers, including general practitioners, nursing homes, and home-patient care, as well as indirect societal costs related to labor absences.

Our study bears several limitations. Besides its retrospective design, a standardized cost matrix was used for calculation and hence equipment costs for laparoscopic and open disposals were not considered on a case-by-case summation. Moreover, averaged costs per domain might have differed between years. This is particularly true during the COVID-19 pandemic when personnel costs were altered due to altering of staff on ICU und operating units. Finally, the learning curve associated with minimally invasive approaches may initially extend operation times [27]. However, as surgical experience grows, both operative time and, subsequently, cost efficiency are likely to improve, thus further supporting the principal conclusion of this study.

## Conclusions

Our results suggest that LTG achieves similar safety and oncological effectiveness compared to OTG, while also presenting favorable economic benefits, though not reaching statistical significance. Notably, a shorter overall hospitalization duration and reduced stays in the ICU emerge as crucial factors in reducing costs within this context. The ongoing establishment of LTG seems therefore not to pose additional financial burdens for surgical departments.

## Electronic supplementary material

Below is the link to the electronic supplementary material.

Supplementary Material 1

## Data Availability

No datasets were generated or analysed during the current study.
