# Supplementary material for: Cost analysis of laparoscopic total versus open total gastrectomy in gastric cancer
Source: Langenbecks Arch Surg. 2025 Jan 8;410(1):30. doi: 10.1007/s00423-024-03562-y (PMC11711782; doi:10.1007/s00423-024-03562-y)
Supplement: Supplementary file 1 — Supplementary Material 1 [file 423_2024_3562_MOESM1_ESM.docx]

**Supplementary material**

**Table 1** Multivariate analysis of factors associated with increased laboratory costs (beyond 75^th^ percentile) of patients who underwent OTG or LTG for EC or cancer of the GEJ

| **Parameters** | **UV** | | | **MV** | |
| --- | --- | --- | --- | --- | --- |
|  | **<2,215 € per case**  **(n = 60)^1^** | **≥2,215 € per case**  **(n = 20)^1^** | ***P^2^*** | **HR (95 % CI)** | ***P^3^*** |
| Male sex | 37 (62%) | 13 (65%) | 0.790 |  |  |
| Age ≥73 years^4^ | 17 (28%) | 6 (30%) | 0.887 |  |  |
| BMI ≥30 kg/m^2^ | 8 (14%) | 4 (20%) | 0.487 |  |  |
| ASA score ≥3 | 23 (38%) | 8 (40%) | 0.895 |  |  |
| CCS ≥11^4^ | 17 (28%) | 8 (40%) | 0.330 |  |  |
| Diabetes | 7 (12%) | 7 (35%) | **0.036** |  | NS |
| Coronary heart disease | 4 (6.7%) | 4 (20%) | 0.102 |  |  |
| Arterial hypertension | 32 (53%) | 12 (60%) | 0.604 |  |  |
| Pulmonary disease | 7 (12%) | 4 (20%) | 0.454 |  |  |
| Neoadjuvant treatment | 57 (97%) | 15 (75%) | **0.010** | 0.02 (0.0-0.27) | **0.005** |
| T stage ≥3 | 31 (52%) | 10 (50%) | 0.897 |  |  |
| Nodal positive disease | 38 (63%) | 10 (50%) | 0.292 |  |  |
| Lymphangiosis carcinomatosa | 13 (22%) | 11 (55%) | **0.005** | 19.8 (4-179) | **0.001** |
| Venous invasion | 2 (3.3%) | 2 (10%) | 0.259 |  |  |
| Length of surgery ≥321 min^4^ | 14 (23%) | 6 (30%) | 0.551 |  |  |
| Need for re-operation | 4 (6.7%) | 4 (20%) | 0.102 |  |  |
| Pneumonia | 5 (8.3%) | 5 (25%) | 0.110 |  |  |
| Anastomotic insufficency | 1 (1.7%) | 4 (20%) | **0.013** |  | NS |
| Anastomotic stenosis | 2 (3.3%) | 1 (5.0%) | 1.00 |  |  |
| Length of ICU stay ≥3 days^4^ | 13 (22%) | 9 (45%) | **0.043** |  | NS |
| Readmission to ICU | 2 (3.3%) | 6 (30%) | **0.003** | 56.1 (3.9-2,024) | **0.008** |
| Length of hospital stay ≥15 days^4^ | 11 (18%) | 10 (50%) | **0.005** |  | NS |
| Major morbidity | 5 (8.3%) | 6 (30%) | **0.024** |  | NS |

^1^ n (%)

^2^ Fisher's exact test; Pearson's Chi-squared test

^3^ Binary logistic regression

^4^ 75^th^ percentile of all patients

UV, univariate analysis; MV, multivariate analysis; BMI, body mass index; ASA, American Society of Anesthesiologists; CCS, Charlson comorbidity score; ICU, intensive care unit; NS, not significant; n/a, not applicable
